# Integrated strategies of support and home care by family caregivers for prevention of hospital readmissions among stroke survivors

**DOI:** 10.1186/s12913-025-13772-9

**Published:** 2025-11-22

**Authors:** Hairui Yu, Jing Yu, Jian Jiao, Ziyue Wang, Ting Luo

**Affiliations:** 1 School of Public Health, University of Alabama, Birmingham, USA; 2 School of Medicine, University of Alabama, 1720 2nd Ave South, Birmingham, AL 35294 USA; 3 School of Nursing and Midwifery, Jiangsu College of Nursing, #9 Keji Rd., Huai’an, Jiangsu 223003 China; 4 Faculty of Humanities and Arts, Macau University of Science and Technology, Avenida Wai Long, Taipa, Macau 999078 China; 5 Faculty of Medicine and Health Sciences, McGill University, 5858 Ch. de la Côte-des-Neiges, 3rd floor, Montréal, QC H3S 1Z1 Canada; 6 Moores Cancer Center, University of California San Diego, 9500 Gilman Dr, La Jolla, CA 92093 USA

**Keywords:** Caregiving, Chinese culture practices, Rehabilitation, Stroke, Qualitative study

## Abstract

**Background:**

Investigation of integrated readmission preventative strategies employed by family caregivers reveals key needs of stroke survivors and their families, yet insufficient feedback limits the ability of healthcare providers to deliver customized and culturally sensitive support. The objective was to identify integrated strategies of Chinese family caregivers to prevent hospital readmissions among stroke survivors.

**Methods:**

Employing a qualitative descriptive study design and utilizing purposive sampling, this research involved ten adult family caregivers who had provided care to a stroke survivor in a community setting for at least six months. Participants were recruited from a tertiary hospital specializing in Chinese medicine that offers a wide range of services. Caregivers were asked questions in a face-to-face, semi-structured interview. The narrative data were analyzed using thematic analysis.

**Results:**

Among the caregivers, seven were female and three were male, with an average age of 55 years. They indicated integrated strategies that fell into six thematic categories reflecting congruence, as a sense of well-being, to reduce hospital readmissions, including: (1) promoting physical activity; (2) integrating pleasurable foods with a balanced diet; (3) monitoring internal and external threats to health and safety; (4) developing individualized motivational strategies; (5) providing emotional support and maintaining optimism; and (6) gaining knowledge from healthcare professionals and fellow caregivers.

**Conclusion and implications:**

Health promotion initiatives might consider integrated strategies and emphasize culturally competent efforts identified in this study. Future longitudinal research on the long-term reduction of readmission risk for stroke survivors is demanded.

**Supplementary Information:**

The online version contains supplementary material available at 10.1186/s12913-025-13772-9.

## Introduction

Stroke is a leading cause of death in China, accounting for more than two million deaths each year [1, 2]. While the authorities have developed national goals aimed at reducing the incidence and mortality rates of stroke and improving the quality of life among stroke survivors [3, 4], the impact of stroke on the Chinese population exceeds the global average [5, 6]. Moreover, this situation is expected to deteriorate further due to factors such as an aging demographic, widespread risk factors including sedentary lifestyles, suboptimal diet quality, hypertension, family history, and poor care both during and post-stroke [7–9].

Adequate post-stroke family care is essential for the long-term survival and quality of life of patients [10, 11]. The frequency of unplanned hospital readmission post-stroke is often used as an indicator of inpatient care quality and reflects poor adherence to post-discharge care regimens [12]. In China, families predominantly take the responsibility for post-discharge care, imposing significant physical, emotional, and financial burdens on caregivers [13]. Informal or family caregivers face a heightened risk of depression extending beyond the acute phase of the stroke [14, 15]. They rely on healthcare professionals for assistance and expertise, as well as on community networks for additional support [16–18]. The lack of coordinated care and adequate home care services in certain regions of China has led to a nearly 20% rate of hospital readmission within three months [19]. Other contributing factors include the declined physical capability of survivors and increased caregiving loads [20], as well as limited financial and community support [7].

Understanding the integrated strategies of support and home care by family caregivers can help researchers and clinicians identify the actions of patients and families and the dynamics of family caregiving for stroke survivors. This is particularly relevant in the context of Chinese cultural practices. For example, increased self-efficacy and provided social support from planning programs can prepare family members for the responsibilities entailed in a smooth transition back home [21, 22], thereby improving the mental and physical well-being of both caregivers and stroke care recipients [22–24]. Prior studies emphasized that health promotion programs designed for Chinese caregivers should consider cultural norms, especially those related to family obligations and self-sacrifice [15, 25], which might influence the decision-making and approaches of caregivers [26].

Nonetheless, the lack of voice from Chinese stroke family caregivers has limited the competency of health professionals and social workers to provide individualized and culturally sensitive support. To fill this gap, the objective of this study is to identify the integrated strategies reported by Chinese family caregivers to prevent hospital readmissions when caring for stroke survivors in community settings.

## Methods

### Setting and participants

This study employed a qualitative research design, utilizing purposive sampling to recruit family caregivers of stroke survivors recently discharged from the rehabilitation department of a comprehensive tertiary Chinese medicine hospital. The hospital is located in Jiangsu Province on the east coast of mainland China. Recruitment criteria for potential participants were as follows: providing care for a stroke survivor in a community setting for six months or longer, never being trained in stroke rehabilitation, being 21 years or older, and speaking Mandarin Chinese to facilitate the interview process. Also, in their recent caregiving of six-month or longer, their care recipients were not diagnosed with severe dysphagia or aphasia or with multiple instances of stroke. Initially, twelve caregivers were interested in the study, but two declined to participate due to scheduling conflicts, resulting in ten interviews with the family caregivers of stroke survivors. Qualitative studies can reach saturation at comparatively small sample sizes (between 9 and 17 interviews), enabling thorough exploration of themes within narrowly defined research objectives [27]. The sample size in this study was considered sufficient, as (a) the purpose of the study was narrowly defined: that is, to identify integrated strategies of Chinese family caregivers to prevent hospital readmissions among stroke survivors, (b) although the majority of our participants were female (70%) and only had 9 years of schooling (70%), our participants varied widely on some other demographic characteristics (e.g., age, employment status) and their characteristics related to their caregiving role (e.g., relationship to the care recipient, duration of care), and (c) our participants provided in-depth information that thoroughly addressed the research question. During the preparation of this manuscript, the authors adhered to the COnsolidated criteria for REporting Qualitative research (COREQ) to ensure transparency and rigor in the reporting of the research methodology, data collection, and findings [28]. Ethical approval was obtained from both the university’s Institutional Review Board and the hospital involved in the study.

### Materials and measures

Family caregivers were asked questions in a single face-to-face, semi-structured interview. To ensure content validity, two steps were completed. First, the interview questions were developed based on a literature review of previous studies on stroke caregiving and Chinese family caregiving [13, 15, 17, 21, 22, 29–31]. This preliminary set of questions in English (See Supplement 1) was then validated by three experts in the field, following which the authors who are bilingual in English and Mandarin Chinese translated the questions to Mandarin and tailored the questions to fit the cultural context. Further, prior to the beginning of the interview, the questions in Chinese were validated by the first author and two physician-scientists from the hospital where the study occurred.

During the interview, caregivers provided demographic details about themselves and the stroke survivors, including age, gender, education level, and employment status. They also discussed the health status of both parties and described the duration of their caregiving experience. Perceived health condition was rated on a 5-point Likert scale including Poor, Fair, Good, Very Good, and Excellent [32, 33]. Caregivers then were asked where their intervention potentially prevented the hospitalization of the care recipient, describing those situations in detail. To facilitate recall and discussion, interview prompts encouraged caregivers to recount specific strategies they employed, such as calling a healthcare provider, administrating medication, changing dietary habits, or assisting with physical activity.

### Data collection

The first author independently led the interviews. Staff from the rehabilitation department and the second author coordinated the interview. Staff from the rehabilitation department identified potential family caregiver participants, inviting them for a conversation with the interviewer. This invitation was extended either in person or via phone, where the interviewer would detail the purpose of the study and the informed consent procedures. Interested caregivers were then scheduled for an interview at a location of their choice, often at their home or in a hospital meeting room. During these meetings, the interviewer clarified any study-related questions, obtained written consent, and conducted the face-to-face interview. These interviews, which were audio-taped for accuracy, typically lasted around one hour or until a comprehensive understanding of the caregiving experience was obtained. To ensure privacy and comfort, interviews were conducted in a private setting with only the interviewer and the participant present.

### Data analysis

Descriptive statistics were applied to the demographic information, and the narrative content underwent a thematic analysis for theme identification, following a conventional approach to identifying categories from the text [34]. The recorded data (i.e., audio-taped recordings) were first transcribed verbatim by the authors, following which the transcripts were translated from Mandarin to English by the first author, who is bilingual in English and Mandarin. The translations were then double-checked for accuracy and corrected through consensus by other members of the research team who are also bilingual in English and Mandarin. In particular, we used an inductive and iterative coding procedure that includes both independent and collaborative work, and the first four authors were involved in the process. The independent work involves each investigator (a) reading each narrative in its entirety to grasp its overall context, (b) extracting statements that describe stroke family caregivers’ strategies to prevent hospital readmissions, (c) assigning open codes to each statement that briefly describes the statement, and (d) clustering open codes into broader themes. The goal of inductive coding is to identify plausible themes from experiences or processes evident in the narratives. Throughout the process, the research team also engaged in collaborative coding and analysis with regular peer debriefing [35].

In particular, collaborative work involves an iterative process of individual and group decision-making where differences in coding and categorizing were openly and thoroughly discussed until a final consensus was reached. The purpose of the collaborative procedure is to reduce individual bias and strengthen the credibility of findings by integrating multiple informed viewpoints [36]. The finalized themes were then synthesized into a comprehensive depiction of caregivers’ strategies, using the process dimensions of Friedemann’s Framework of Systemic Organization (FFSO) to structure the findings [37, 38].

The FFSO postulates that congruence is the “energy flowing freely between systems that are compatible in patterns and rhythms and attuned to each other.” Humans experience the presence of congruence as a sense of well-being or health, while incongruence refers to negative feelings or illness/disease [37, 38]. The current study examines how Chinese family caregivers and stroke survivors endeavor to achieve and maintain congruence through four process dimensions: system maintenance, system change, coherence, and individuation.

System maintenance and change are two critical dimensions. System maintenance consists of roles, rules, organizational patterns, rituals, decision-making, and division of labor rooted in tradition, which could be used early on in caring by family caregivers to sustain the lives of stroke survivors [37, 38]. On the other hand, system change means significant alterations of day-to-day operations that lead to shifts in the traditional value system over time. True change incorporates and channels new information, tests values and adjusts beliefs, adapts behavior patterns to concur with new values, and incorporates new patterns by changing or eliminating old ones as caregivers continue caring [37, 38].

Coherence and individuation represent the relational and developmental aspects of the caregiving experience. Coherence is defined as emotional bonding and caring relationships, such as those between stroke caregivers and their care recipients, facilitating mutual support. Individuation refers to enhancing individual learning, changing attitudes, incorporating information, and sharing opinions and beliefs. This process of individuation occurs while learning how to deal with the effects of the stroke on caregivers and survivors [37, 38].

## Results

### Demographic characteristics of caregivers and stroke survivors

Table 1 displays detailed demographic information for both the caregivers and stroke survivors. Among the caregiver participants, ages ranged from 23 to 85 years, with a majority being female (70%) and lacking a high school diploma (70%). Their family relationships were spouse (40%), son (30%), daughter (20%), and granddaughter (10%). More than half (60%) had been providing care for over a year. Eight (80%) of the participants reported not being employed, with multiple indicating they had retired after their care recipient’s stroke, believing that the stroke survivor required more assistance than they would provide while working. Most of the participants reported their health as fair (80%).

The ages of the stroke survivors spanned from 52 to 90 years, with a majority being male (70%). According to the participating caregivers, the health of the care recipients was good (40%), fair (20%), or poor (40%) at the time of the interview.

**Table 1:** Demographic characteristics of caregivers and care recipients

	Caregiver (CG)							Care Recipient (CR)			
#	Pseudonym^	Age	Gender	Education Level (Years of Schooling)	Employment Status	CG’s Perception of Own Health *	Relationship to CR / Duration of Care	Pseudonym^	Age	Gender	CG’s Perception of CR’s Health *
1	Jun	23	Female	16 years	Not employed	Fair	Granddaughter / < 1 Year	Ou	76	Male	Poor
2	Lin	40	Female	12 years	Self employed	Fair	Daughter / 5–6 Years	Bo	66	Female	Poor
3	Hai	48	Male	12 years	Self employed	Fair	Son / < 1 Year	Fang	76	Male	Good
4	Zhi	58	Male	9 years	Retired	Fair	Son / 2–3 Years	Ei	80	Male	Poor
5	Shun	85	Female	9 years	Retired	Good	Wife / >10 years	Ai	90	Male	Good
6	Cheng	70	Female	9 years	Retired	Fair	Daughter /1year	Leng	90	Female	Fair
7	Yi	62	Female	9 years	Retired	Good	Wife / < 1 Year	In	65	Male	Poor
8	Ke	51	Male	9 years	Not employed	Fair	Husband/ 1 year	Geng	52	Female	Good
9	Xue	70	Female	9 years	Retired	Fair	Wife / < 1 Year	Ming	76	Male	Good
10	Neng	40	Female	9 years	Retired	Fair	Daughter / 5–6 years	Dong	75	Male	Fair

* Scale of the perceived health: Poor, Fair, Good, Very Good, and Excellent

^ Pseudonymization for de-identifying personal data

### Themes

Table 2 presents quotes illustrating six themes derived from the experiences of family caregivers and stroke survivors, focusing on their strategies to potentially prevent hospital readmissions of the survivors. These themes highlight a progression toward congruence, signifying health and well-being, framed with the conceptualization of four process dimensions of FFSO: system maintenance, system change, individuation, and coherence.

**Table 2:** Six themes for caregiver strategies with exemplary quotes

**1. Promoting physical activity (system maintenance)**
*“At the beginning*,* his feet and hands could not move…I told him that you must move*,* you must move*,* do massage*,* massage your hands and feet… I have always encouraged rehabilitation*,* though I know he might fear it since rehabilitation is painful. I sometimes had to push him to move. But he was sometimes resistant*,* and he complained pain when doing so.” (#1 Jun*,* age 23*,* granddaughter; Ou*,* age 76)*
*“We usually walk every day. Previously her left body could not move*,* but we kept encouraging her to exercise. Now*,* she can move her entire body…We taught her self-massage*,* taught her to use her stronger hand to help another hand*,* and then lift it up.” (#8 Ke*,* age 51*,* husband; Geng*,* age 52).*
*“I support his knees with my knees*,* and then letting him use his strength to stand*,* which aimed to increase his recovery speed.” (#3 Hai*,* age 48*,* son; Fang*,* age 76)*
*“In addition to the fitness bike*,* I bought and purchased other equipment. The fitness bike can exercise both hands and legs.” (#3 Hai*,* age 48*,* son; Fang*,* age 76)*
*“He was sometimes reluctant to go*,* but I helped him. I never gave him a wheelchair. Once he is in a wheelchair*,* he would not want to get up. Without a wheelchair*,* he would be forced to walk.” (#5 Shun*,* age 85*,* wife; Ai*,* age 90).*
**2. Integrating pleasurable foods with a balanced diet (*system maintenance*** **and** ***change***, ***individuation*)**
*“He likes to eat pork and chicken*,* but I know he should avoid greasy stuff. I also wanted to eat meat at the time*,* so I bought it for him. My dad gets him some nutritional supplements*,* while my grandmother buys a lot of vegetables.” (#1 Jun*,* age 23*,* granddaughter; Ou*,* age 76)*
*“We cook a small amount of meat when we cook at home*,* and then it is very light. There is very little salt and [we] keep the ingredients fresh. We pay attention to the diet.” (#10 Neng*,* age 40*,* son; Dong*,* age 75)*
*“Being his children*,* not serving him a big meal would seem unfilial. However*,* if we don’t control his diet*,* he is at risk of gaining weight*,* which could lead to higher blood pressure. We feel bad about greasy and meaty food*,* but he likes to eat them. If we don’t give him meat*,* his mood would change… Without his favorite dishes*,* he might become unhappy*,* reminding us constantly.” (#4 Zhi*,* age 58*,* son; Ei*,* age 80)*
*“We are cautious about his meat intake*,* opting for shredded pork instead. The kids suspect it’s to save money*,* but it’s really because he shouldn’t consume much meat.” (#9 Xue*,* age 70*,* wife; Ming*,* age 76)*
“*I bought him some shrimp (six dollars) today*,* although it was not cheap. To maintain his health*,* we are willing to do so”.* (#7 Yi, age 62, wife; In, age 65)
**3. Monitoring internal and external threats to health and safety (*system maintenance*** **and** ***change*)**
*“We usually help her to measure her blood pressure*,* determine that the blood pressure is within a reasonable range*,* and give her a blood pressure test three or four times a day.” (#2 Lin*,* age 40*,* daughter; Bo*,* age 66)*
*“When he stays with me*,* I encourage him to climb stairs*,* always with a focus on his safety.” (#3 Hai*,* age 48*,* son; Fang*,* age 76)*
*“I often touch his hand or forehead to see if he has a fever. When we are feeding him*,* we are very careful and afraid to burn him…” (#7 Yi*,* age 62*,* wife; In*,* age 65)*
*“She is sometimes lazy and doesn’t want to take medicine*,* but I ensure she does.” (#8 Ke*,* age 51*,* husband; Geng*,* age 52)*
*“In certain weather conditions*,* like rain or strong winds*,* we advise him against going for walks. But if he insists on going out*,* we can’t stop him. We suggest wearing less clothing in the heat and more when it’s cold.” (#4 Zhi*,* age 58*,* son; Ei*,* age 80)*
*“Even though she become noisy at night*,* we look after her…she is sick. She lost her memory and lost some consciousness. We believe as long as she lives*,* it’s our duty to care for her each day.” (#6 Cheng*,* age 70*,* daughter; Leng*,* age 90)*
**4. Developing individualized motivational strategies (** ***individuation and system change*** **)**
*“He likes watching TV. When I watch TV with him*,* I discuss TV programs with him and encourage him to speak.” (#1 Jun*,* age 23*,* granddaughter; Ou*,* age 76)*
*“She suffers from pain and feels that she is going to die. I tell her that for others*,* her passing would merely be news*,* but for her*,* it means losing everything. For the family*,* because we are children*,* it is normal for the young to bury the elderly*,* but your sisters*,* if you don’t want to live*,* what might they think about.” (#2 Lin*,* age 40*,* daughter; Bo*,* age 66)*
*“I stimulate her to speak more often. Sometimes when she doesn’t want to talk*,* I might yell at her*,* give her some excitement*,* and she realize what she should say… She was depressed and very annoyed and impatient… my daughter just got married*,* I told her that she will have a grandson in the future and we need her … to encourage her” (#8 Ke*,* age 51*,* husband; Geng*,* age 52)*
*“He doesn’t like to eat fruit. But if you don’t eat fruit*,* you will be constipated. This causes a family disagreement as we tried to persuade him to eat fruit. After some resistance*,* he began developing the habit of eating fruit.” (#10 Neng*,* age 40*,* son; Dong*,* age 75)*
*“My child is fifteen years old now and in junior high school. She cherishes this child. I said*,* if you don’t commit to rehabilitation*,* you can’t get out of bed*,* and then I have to care of you exclusively. Then I don’t have time to take care of the child*,* so I might neglect our child. The child has average grades and needs to be supervised. She knows this and works harder to recover.” (#2 Lin*,* age 40*,* daughter; Bo*,* age 66)*
**5. Providing emotional support and maintaining optimism (** ***coherence*** **)**
*“If I were to complain every day*,* life would be very bad. My mom used to be in a bad mood and I would think about how to improve her mental condition. My mother sometimes would use hurtful words*,* which could make others angry.” (#2 Lin*,* age 40*,* daughter; Bo*,* age 66)*
*“The little things in life will be very funny… For example*,* [telling him about] a relative who has had a cute baby… We might choose not to tell him [the patient] that he is seriously ill and we prevent him from knowing his condition… I don’t think he knows what his condition exactly is and avoid discuss it in his presence.” (#1 Jun*,* age 23*,* granddaughter; Ou*,* age 76)*
*“I have always maintained my optimism and health. I know that one day*,* he will leave. But I want to accompany him to the last day. If I am in a bad mood*,* it will affect his mood. He will also care about my mood.” (#5 Shun*,* age 85*,* wife; Ai*,* age 90)*
*“If I have time*,* I will take her to the park or by the river. I take her to relax*,* and then talk to her about the good things.” (#8 Ke*,* age 51*,* husband; Geng*,* age 52)*
*“He wants to talk but forgets what he was going to say as soon as he starts talking. Then I teach him to talk. I teach him what he should say and he can follow. We all felt that he was very unhappy at the time*,* but it is getting a lot better now.” (#9 Xue*,* age 70*,* wife; Ming*,* age 76)*
**6. Gaining knowledge from healthcare professionals and fellow caregivers (** ***individuation*** **)**
*“The doctor told us how to pay attention to his diet and medication. In this way*,* we gradually learned how to take care of him.” (#5 Shun*,* age 85*,* wife; Ai*,* age 90)*
*“I felt that his condition was not right*,* but I didn’t know what to do at the time*,* and I didn’t know what went wrong. After a while*,* I found that his hands and feet were cold and purple*,* and I was very scared. I then called the doctor to come over and found that his blood sugar was very low*,* reading only three or five points. He experienced cramps and vomiting but recovered after receiving emergency care.” (#7 Yi*,* age 62*,* wife; In*,* age 65)*
*“When the physical therapists practice the rehab approaches or moves in the rehab department*,* we watch and learn. Then we repeat at home*,* to help him recover.” (#10 Neng*,* age 40*,* son; Dong*,* age 75)*
*“A friend’s family member has such a disease. We exchange information and experiences. Sometimes*,* at the hospital … the patient’s family also share some their knowledge. We learn from each other…” (#3 Hai*,* age 48*,* son; Fang*,* age 76)*
*“We have very little communication. We’re aware that some neighbors in our residential building complex have experienced strokes. However*,* in fact*,* their families are often very busy with work. They need to earn a living*,* and they have no time.” (#1 Jun*,* age 23*,* granddaughter; Ou*,* age 76)*

Note. * Quotes from caregivers that illustrate this theme are labeled with information on the caregiver and care recipient *(#X caregiver’s pseudonym*,* age relationship to care recipient; care recipient’s pseudonym*,* age*), as outlined in the demographic Table 1

#### Promoting physical activity

Caregivers highlighted the importance of daily movement and active participation in rehabilitative exercises for their care recipients. They encouraged practices like self-massage and using their unaffected hand to assist the other. While caregivers recognized that rehabilitation could be challenging and sometimes met with resistance, they viewed continued participation as an essential component of recovery. In addition to being emotionally invested, the participants were physically and financially committed to the rehabilitation process. They provided physical support, such as stabilizing the care recipients’ knees with their own or stimulating their hands through gentle rubbing. Furthermore, they purchased therapeutic devices or rehabilitative equipment (e.g., fitness bikes) on their own to use with their care recipient at home. This theme reflects the dimension of system maintenance, showcasing efforts to help their care recipients maintain their lives before the stroke and recover lost functionalities.

#### Integrating pleasurable foods with a balanced diet

Participants emphasized the importance of a healthy diet for their care recipients, carefully considering which foods were beneficial and which should be avoided. Participants recognized the significance of incorporating cultural options and comforting foods into their diet. Caregivers ensured that the daily diet included foods that their care recipients had always enjoyed, demonstrating an aspect of *system maintenance.* Simultaneously, they adapted by reducing unhealthy foods or altering cooking methods to make them healthier, such as less salt and fresher ingredients, reflecting elements of system *change and individuation*.

#### Monitoring internal and external threats to health and safety

Participants shared the constant vigilance that they practiced with regard to their care recipient. They reported careful monitoring of their care recipients’ physical health on a daily basis (e.g., body temperature, blood pressure, medications taken). They were also attentive to the current situation and aware of the surrounding environment to safeguard their care recipients from potential harm and prevent any new injuries (e.g., falling on the stairs). This theme illustrates the commitments of caregivers to both maintaining the pre-existing lifestyle of their care recipients and adapting practices as necessary to avert further health deterioration or injury, embodying the process dimensions of *system maintenance* and *change*.

#### Developing individualized motivational strategies

Participants used personalized strategies that they thought would motivate their care recipients, even at the risk of momentary displeasure. Tactics included leveraging feelings of guilt and invoking a sense of family obligation. They encouraged care recipients to work harder in their recovery by focusing on the future of the family and younger family members (e.g., a child may not do well in school if the family focus is on the care recipient). They emphasized the necessity for the care recipients to regain health to support raising children and grandchildren. Some study participants actively encourage care recipients to communicate or use enjoyable activities, such as watching a favorite television show, as a means to stimulate conversation. These methods reflect caregivers’ understanding of what led to positive, long-term results for their specific care recipient, illustrating elements of i*ndividuation* and *system change*.

#### Providing emotional support and maintaining optimism

Caregivers dedicated themselves to providing emotional support and establishing a meaningful connection with their care recipients. They understood the importance of positive mental health and found ways to promote it. Participants said they often told funny stories or shared the good news about the family to make care recipients smile and laugh. The participants also felt it was critical to remain optimistic about the situation and the care recipient’s future. This theme illustrates the process dimension of *coherence*, highlighting the emotional bonds between caregivers and care recipients through shared optimistic conversations and enjoyable moments.

#### Gaining knowledge from healthcare professionals and fellow caregivers

Participants actively sought information and guidance from healthcare professionals, yet they expressed a desire for more opportunities for support from fellow caregivers and the community. Although they asked for answers from physicians and rehabilitation therapists, there was a strong interest in exchanging insights and discovering solutions to caregiving challenges with others who had faced similar situations. They also wanted greater emotional support from their caregiving peers. The proactive approach to acquiring new knowledge and refining caregiving skills illustrates the process dimension of the *individuation*.

## Discussion

The current study provides a voice to family caregivers in mainland China, offering a rich description of their experiences from their perspectives. The study focuses on integrated strategies used by caregivers to prevent hospital readmissions, underscoring their positive effects on the health and well-being of their care recipients. We identified six key emerging themes from caregivers’ strategies, serving as foundations for effective health promotion and hospital readmission reduction efforts. These themes were organized within the process dimensions of FFSO, providing a structured and theoretical basis for understanding these strategies [37, 38].

This study found that Chinese family caregivers played significant roles in supporting daily activities and maintaining the health of stroke survivors, while also working to reduce hospital readmission rates. This aligned with previous research on possible strategies for reducing readmissions significantly with family support when available [39, 40]. Researchers have reported that caregiver burden is most common when the stroke patient has lower physical functioning and requires extensive care [15]. Consequently, family caregivers often encourage care recipients to remain active in an attempt to prevent further deterioration and dependency [14]. The caregivers in the current study expressed an understanding of their role in adapting activities and behaviors to improve functionality, although they sometimes faced challenges in motivating older stroke patients to engage in physical activities. The findings provide insight into the complex dynamics of supporting family caregivers in their efforts to involve care recipients in physical therapy and rehabilitation activities.

High hospital readmission rates in China suggest that many caregivers and stroke survivors may face difficulties in effectively managing stroke conditions [19]. Certain traditional Chinese diets, which often include higher levels of salt and in some regions are unbalanced (e.g., relatively lower consumption of fiber), are potential contributors to the higher prevalence of both hypertension and stroke in China [7]. Consistent with previous research, the current study underscores the critical influence of cultural values and filial piety on caregiving choices [13, 25]. Within Chinese culture, not providing meat to older adults is often viewed as an act of disrespect, potentially leading to criticism from relatives and society. This context significantly influences caregivers’ approaches to modifying everyday routines, including diet and food choices [13, 25]. Caregivers in this study conveyed the necessity of a balanced diet and appropriate nutrition for post-stroke care, while also wanting to respect their elder relatives’ preferences for traditional, comforting, and enjoyable foods. This finding suggests a potential avenue for future research to explore the impact of cultural factors on dietary adherence and the strategies employed by caregivers to effectively address this challenge.

Family members involved in this study cited several metrics used to monitor the health of the stroke survivor including normal temperature and blood pressure checks, medication management, and attention to safety concerns such as the temperature of food and safe use of stairs. Consistent with previous studies, taking antihypertensive medications was more likely to continue in older patients and patients with comorbidities [41, 42]. Furthermore, receiving social support from providers such as spouses/partners, children, and relatives plays a role in enhancing adherence to antihypertensive medication among patients with cardiovascular issues in community settings [43].

The study found that more than half (70%) of caregivers had less than a high school education. However, research shows that educational initiatives are crucial in preparing families for a smooth transition from hospital to home care, thereby enhancing the health and well-being of both stroke survivors and their caregivers [21, 23, 29]. Despite their limited formal education, participants effectively leverage tools and technology to maintain the health of their family members, yet they also expressed a desire for ongoing information and support. Mobile technology used in family caregiving to find information, access resources, and interact with others, has been shown to improve the quality of long-term care [44]. Furthermore, mobile health technologies not only help mitigate isolation but also deliver therapeutic interventions and track the health indicators of individuals with disabilities [45, 46]. Understanding the specific needs of family members, especially those with lower levels of education and skills, may aid in the development of programs aimed at addressing disparities in stroke care management.

This study also found that caregivers integrated individualized and sometimes creative strategies to encourage physical activity and healthy eating behaviors. Some caregivers described using persuasive approaches that involved emotional appeals or strong verbal encouragement to motivate patients in their recovery. While these methods may stem from good intentions, they risk creating unintended stress. More collaborative and patient-centered communication, as exemplified by shared goal-setting, can be both more satisfactory and more effective in promoting long-term engagement. Moreover, these approaches varied widely and were tailored to the unique motivational drivers of each care recipient, and deeply rooted in cultural expectations of family and the concept of familism. Research shows that interventions aimed at preparing family members to assume post-discharge stroke care are most effective when they are individualized to the needs and abilities of the care recipients [22]. The evidence that family members seek innovative ways to encourage compliance with stroke care protocols underscores the importance of individualized interventions. Despite the challenges of stroke care, participants in this study were dedicated to fostering moments of happiness and nurturing personal connections. Similar to findings in prior studies, caregivers found ways to strengthen family bonds and cultivate close relationships with their family members, even in the face of reduced physical and cognitive functions [47].

Finally, the current study adds to the literature highlighting the urgent need for enhanced support systems for family stroke caregivers. These caregivers actively seek information from healthcare providers regarding the health and care of the stroke survivor, but often face challenges due to inconsistent advice and difficulties in accessing information. These challenges may explain why caregivers increasingly value peer support from fellow caregivers, as it offers practical and relatable insights. A study of Chinese stroke caregivers’ needs during the COVID-19 pandemic illuminated the struggle family members experience when support and education options are limited and encouraged an increase in available options [24]. While the study was conducted during an unprecedented time of isolation, it supports the need for emerging technologies such as telemedicine for family members in need of support and information [24]. Additionally, participants expressed a stronger desire for more opportunities to connect with others in similar situations, indicating potential avenues for enhancing support and fostering community among family caregivers of stroke survivors.

This study represents one of the few investigations into strategies employed by Chinese family caregivers of stroke survivors to minimize hospital readmission. To our knowledge, this is the first to delve into this topic with such depth and detail. The insights garnered on the strategies taken by caregivers could enhance our understanding of the specific desire, design, and implementation of culturally sensitive health programs tailored for Chinese family caregivers. Guiding with systemic organization, this research identified six key themes that underscore the positive impact of family caregivers on the health and well-being of their recipients.

There are several limitations of this study. The health status of both caregivers and care recipients was self-described, which could introduce bias and differ from what might be revealed through clinical assessments such as motor function scales. Additionally, there was no mechanism for follow-up with participants to elaborate on their responses, which could impact the clarity of the findings. While the study identifies conceptual relationships between adaptive processes and outcomes such as reduced readmissions, it does not establish causality. These limitations may affect the generalizability of the findings, and it is up to the reader to determine if the findings apply to another context. Future studies may consider recruiting a larger sample or using different sampling methods to explore the strategies used in preventing hospital readmissions among stroke survivors.

## Conclusion

In this study, stroke caregivers and survivors described integrated strategies aimed at preventing hospital readmissions and return to their pre-stroke lives (*system maintenance*) by promoting physical activity and monitoring the survivors’ health and safety, and adapting to life changes post-stroke (*system change*) by making compromises between healthy living and cultural preferences. They sought support from healthcare professionals and fellow caregivers (*coherence*), fostered optimism by engaging survivors in meaningful social connections (*coherence*), and developed innovative motivational strategies to navigate their new circumstances post-stroke (*individuation*) with their care recipients.

The six themes derived from the process dimensions of FFSO framework may offer a structured lens for understanding post-stroke adaptation. This conceptual model suggests that integrated support programs need to move beyond one-size-fits-all advice, thus expectedly benefiting the health and congruence of caregivers and their care recipients. This approach provides a potential avenue to ensure smooth adaptation and maintain care quality and continuity in stroke care management.

Health promotion initiatives such as remote or in-person educational programs for stroke survivors and their caregivers might consider integrating and emphasizing culturally competent efforts identified in this study, including engaging in physical activity, maintaining a balanced diet, preventing injuries, providing emotional support, and employing customized strategies. Future longitudinal research may establish which components of this integrated model most effectively reduce long-term readmission risk for stroke survivors.

## Supplementary Information

Below is the link to the electronic supplementary material.

Supplementary Material 1

## Data Availability

The datasets generated and/or analyzed during the current study are available from the corresponding author(s) upon reasonable request.

## Abbreviations

FFSO: Friedemann’s Framework of Systemic Organization
