# Supplementary material for: Integrated strategies of support and home care by family caregivers for prevention of hospital readmissions among stroke survivors
Source: BMC Health Serv Res. 2025 Nov 22;25:1628. doi: 10.1186/s12913-025-13772-9 (PMC12752098; doi:10.1186/s12913-025-13772-9)
Supplement: Supplementary file 1 — Supplementary Material 1 [file 12913_2025_13772_MOESM1_ESM.docx]

***First, I am going to ask you some questions about you.***

1. How old are you? _________ YEARS OLD
2. ONLY ASK IF UNKNOWN: What is his/her gender? MALE FEMALE

1 2

1. What is the highest level of education you have completed?

1 LESS THAN HIGH SCHOOL

2 hIGH SCHOOL GRADUATE

3 SOME COLLEGE

4 ASSOCIATE DEGREE

5 COLLEGE GRADUATE

6 POST GRADUATE DEGREE

4) Are you currently employed?

Full-time Part-time Medical leave Not employed 3 2 1 0

a) What is/was your (paid) occupation? _____________________________________________

5) In general, would you say your health is?

Poor Fair Good Very Good Excellent

0 1 2 3 4

#### *Next, I am going to ask you some questions about the person you are caring for with stroke.*

1. Did he or she have an inpatient stroke rehabilitation stay?

YES NO

1 2

2) What is your relationship to the person for whom you provide care? Your care recipient is your . . .

1 husband

2 wife

3 mother

4 father

5 Other relative, Specify: ________________________________________________

3) When was your [care recipient] discharged from the initial hospital stay for stroke?

_______________________MONTH/YEAR (IF < 6 MONTHS AGO, CAREGIVER IS NOT ELIGIBLE.)

1. How long have you been providing care for your [care recipient]? _________ MONTHS
2. What is his/her age? _________ YEARS OLD

6) ONLY ASK IF UNKNOWN: What is his/her gender? MALE FEMALE

1. 2

7) Where does your [care recipient] live?

1. ALONE IN HIS/HER OWN HOUSE
2. WITH OTHERS IN A HOME
3. WITH ME (CAREGIVER)
4. REHABILITATION DEPARTMENT IN A HOSPITAL
5. LONG TERM CARE FACILITY
6. OTHER, Specify: ______________________________________________

8) In general, would you say the health of your [care recipient] is?

Poor Fair Good Very Good Excellent

0 1 2 3 4

9) In addition to the stroke, does he/she have other chronic medical conditions (e.g. diabetes, heart disease)?

NO YES

0 1 a) IF YES, What are they? ___________________________________________

____________________________________________________________________________________________________

#### TURN ON AUDIO RECORDER. *Now I am going to ask you some questions about how your [care recipient] has been doing since being discharged from the hospital for stroke, that is since* [MONTH/YEAR].

1. I want you to think back to whether there have been times since your [care recipient’s] discharge that you’ve thought that if you did not take action he/she would need to go back to the hospital.

Tell me your story of what happened during that time / those times.

IF THE CAREGIVER SAYS HE OR SHE CANNOT THINK OF A TIME, USE THE FOLLOWING PROMPTS:

For example, did you call a healthcare provider, give medication to your [care recipient], ask a friend for advice, or search the internet for health information.

IF HIS OR HER “STORY” DOES NOT INCLUDE ALL THE INFORMATION WANTED, THEN ASK:

What actions did you take to try to prevent your [care recipient] from being readmitted to the hospital?

Why did you choose that action? OR Why did you think that would help?

Did your [care recipient] end up going to the hospital?

******************************************************************************************

NOTES:

1. Action:
2. Reason:
3. Has your [care recipient] been readmitted to any hospital in the last six months?

NO YES

- - 1. If YES, how many times? ____________

FOR EACH VISIT, ASK . . .

1. What was the reason?

ii) What was the reason?

1. Is there anything else you would like to add?

Thank you very much for your help.
